# Factors Associated with Falls in Community-Dwelling Older People with Focus on Participation in Sport Organizations: The Japan Gerontological Evaluation Study Project

**DOI:** 10.1155/2014/537614

**Published:** 2014-05-13

**Authors:** Takahiro Hayashi, Katsunori Kondo, Kayo Suzuki, Minoru Yamada, Daisuke Matsumoto

**Affiliations:** ^1^Center for Well-Being and Society, Nihon Fukushi University, 5-22-35 Chiyoda, Naka-ku, Nagoya, Aichi 450-0003, Japan; ^2^Department of Physical Therapy, Tokai College of Medical Science, 2-7-2 Meiekiminami, Nakamura-ku, Nagoya, Aichi 450-0003, Japan; ^3^Department of Policy Studies, Aichi Gakuin University, 12 Araike, Iwasaki-cho, Nisshin, Aichi 470-0131, Japan; ^4^Department of Human Health Sciences, Graduate School of Medicine, Kyoto University, 53 Iwasaki-cho, Shogoin, Sakyo-ku, Kyoto, Kyoto Prefecture 606-8507, Japan; ^5^Department of Physical Therapy, Faculty of Health Science, Kio University, 4-2-2 Umaminaka, Koryo-cho, Kitakatsuragigun, Nara 635-0832, Japan

## Abstract

*Objective.* Promoting participation in sport organizations may be a population strategy for preventing falls in older people. In this study, we examined whether participation in sport organizations is associated with fewer falls in older people even after adjusting for multiple individual and environmental factors. *Methods.* We used the Japan Gerontological Evaluation Study data of 90,610 people (31 municipalities) who were not eligible for public long-term care. Logistic regression analysis was performed, with multiple falls over the past year as the dependent variable and participation in a sport organization as the independent variable, controlling for 13 factors. These included individual factors related to falls, such as age and sex, and environmental factors such as population density of the habitable area. *Results.* A total of 6,391 subjects (7.1%) had a history of multiple falls. Despite controlling for 13 variables, those who participated in a sport organization at least once a week were approximately ≥20% less likely to fall than those who did not participate at all (once a week; odds ratio = 0.82 and 95% confidence interval = 0.72–0.95). *Conclusion.* Participation in a sport organization at least once per week might help prevent falls in the community-dwelling older people.

## 1. Introduction

Prevention of falls in older people is a major global public health concern [1]. It has been reported that, in older people ≥65 years of age, 1 in 3 falls at least once a year. Injuries from falls can also lead to reduced physical function [2–4]. In Japan, the nation with the highest proportion of older citizens [5], falls and fractures are one of 10 cases requiring long-term care among older people aged ≥65. They are among the five main causes for older people's long-term care [6].

Previous studies have reported several factors associated with the risk of falling, including old age [7], female gender [8, 9], history of falls [10, 11], vision problems [12], depression [13, 14], and reduced strength and balance [15]. It has also been shown that increasing physical activity through exercise interventions that include strength and balance training can prevent falls [3, 16, 17]. However, fall-prevention programs that focus on strength and balance training have poor cost-effectiveness [18]. Since 2006, many municipalities in Japan have spent large amounts of money on fall-prevention programs using high-risk approaches intended for early identification of high-risk individuals. However, numerous issues have been exposed, such as the cost of screening subjects and the fact that few subjects participate in the recommended programs after being screened. There have thus been calls to switch from a high-risk strategy to a population strategy.

Therefore, this study focused on physical activities that community-dwelling older people can easily participate in during their everyday lives as a population approach; specifically, we analyzed the relationship between participation in community sport organizations and the incidence of falls. Participation in sport organizations is linked to fall prevention through direct effects such as increase in physical activity and improving strength and balance [19, 20]. Participation in sport organizations is also thought to indirectly protect health by providing social support and through social networks [21]. Previous studies have found that participation in sport organizations reduces the risk of dementia [22] and stroke [23], as well as reducing functional decline [24]. Therefore, the incidence of falls in older people who participate in nearby sport organizations would be lower than in those who do not. However, to our knowledge, no studies have verified this connection.

The purpose of this study was to verify if population strategies for promoting participation in sport organizations prevent falls in community-dwelling older people and to examine whether the connection between fewer falls and participation in sport organizations among this population is evident after controlling for multiple individual and environmental factors, particularly depression and motor abilities.

## 2. Materials and Methods

### 2.1. Study Sample

The present analysis was based on a subset of the Japan Gerontological Evaluation Study (JAGES) project date. The JAGES project is an ongoing prospective cohort study investigating factors associated with the loss of health related to functional decline or cognitive impairment among individuals aged ≥65 years. In 2010-2011, responses were received by post from 112,123 people in 31 municipalities across Japan (response rate: 66.3%). We further excluded those who did not provide information on age or sex (*n* = 12,627) and history of falls (*n* = 4,527), and those who needed assistance in activities of daily living (ADL) (*n* = 4,359). Therefore, the current study population consisted of 90,610 subjects. If the respondents did not respond to other variables, the corresponding observations were assigned to the “missing” category.

Ethical approval for the study was obtained from the Nihon Fukushi University Ethics Committee.

### 2.2. Outcome Variable

History of falls was ascertained by asking, “Have you had any falls over the past year?” with possible answers of “multiple times,” “once,” or “none.” Multiple falls were used as an outcome (the incidence of falls), and the last 2 categories were combined because previous studies have found that single fallers are more similar to nonfallers than to recurrent fallers on a wide range of medical and physical risk factors [25, 26].

### 2.3. Main Predictors

Participation in a sport organization was ascertained by asking, “How often do you participate in a sport group or club?” with possible answers of “almost every day,” “2 or 3 times a week,” “once a week,” “once or twice a month,” “several times a year,” and “I do not engage in any sport activities,” and was categorized into the following 6 groups: “almost every day,” “2 or 3 times a week,” “once a week,” “once a month or less,” “not participating,” and “missing.” We defined participation in a sport organization as participation in a sport group or club in the community base in this study.

### 2.4. Covariates

On the basis of the results of previous studies [9, 15, 27–29], we selected the covariates that may correlate with falls. First, we used the basic characteristics such as age, sex, and socioeconomic status (educational attainment and annual equivalent income). Educational attainment was ascertained by asking, “How many years of formal education have you had?” with possible answers of “less than 6 years,” “6–9 years,” “10–12 years,” “more than 13 years,” and “other.” We divided the responses into 4 groups: “less than 6 years,” “6–9 years,” “10–12 years,” “more than 13 years,” and we included “other” in the same category as “missing” [28, 30]. To adjust household income for household size, the annual equivalent income was calculated by dividing the household income by the square root of the number of household members and was categorized into 3 groups: ≥2.5 million yen (high), less than 1.5–2.5 million yen (middle), and <1.5 million yen (low).

Physical traits used included current medical history related to falls (stroke, osteoporosis, joint disease/neuralgia, injury/fracture, mental illness, impaired vision, and impaired hearing) and physical ability. Physical ability was ascertained by asking, “Do you go upstairs without holding on to the handrail or the wall?” and “Do you get up out of a chair without holding anything?” with possible answers of “Yes” or “No.” Depression was assessed with the short version of the Geriatric Depression Scale-15 developed for self-administration in the community using a simple yes/no format [31] and was categorized into 3 groups: 0–4 (no), 5–9 (mild), and 10–15 (moderate to severe) [32].

For lifestyle habits, we used walking in minutes/day and frequency of outings. Walking in minutes/day was categorized into 2 groups: <30 minutes (low) and ≥30 minutes (high) [33]. Frequency of outings was categorized into 3 groups: almost every day, 2-3 times a week, and once a week or less [28].

For environmental characteristics, we used the surrounding environment and population density variation. The surrounding environment was ascertained by asking, “Are parks or foot paths suitable for exercise or walking within 1 km from your home?” and “Are locations difficult for walking, such as hills or steps, within 1 km from your home?” with possible answers of “many,” “some,” “few,” and “none.” Both items were categorized into 2 groups: “many” or “some” (Yes) and “few” or “no” (No), and we included “other” in the same category as “missing.” In order to determine whether geographical positioning was a significant factor, the 31 municipalities were classified into 3 groups: urban (densities over 1,500 people per square kilometer), semiurban (densities between 1,000 and 1,500 people per square kilometer), and rural (densities below 1,000 people per square kilometer) [34].

### 2.5. Statistical Analysis

We performed logistic regression to examine the association between participation in a sports organization and the incidence of falls. We calculated the odds ratios (OR) and 95% confidence intervals (95% CI) for the incidence of falls. First, the univariate ORs were calculated for participation in a sport and each covariate (crude OR). Second, 3 logistic regression models were constructed. In model 1, age, sex, educational attainment, and annual equivalent income were added to the univariate models for participation in a sport organization to adjust for sociodemographics. In model 2, current medical history related to falls (stroke, osteoporosis, joint disease/neuralgia, injury/fracture, mental illness, impaired vision, and impaired hearing), physical ability, depression, walking in minutes/day, and frequency of outings were added to model 1 to adjust for sociodemographics. In model 3, surrounding environment and population density variation were added to model 2 to adjust for sociodemographics. All statistical analyses were conducted using IBM SPSS statistical software Ver.21 (IBM Corp.).

## 3. Results

Table 1 shows the demographic and health characteristics of all study respondents. These comprised 41,912 men (46.3%) and 48,698 women (53.7%), and the mean age was 73.9 ± 6.1 years. A total of 6,391 (7.1%) of the 90,610 respondents reported the incidence of falls.

### 3.1. Frequency of Participation in Sport Organizations and Fall Incidence

Table 2 shows the frequency of participation in a sport organization. There were 53,645 subjects (59.2%) in the not participating group, 7,020 subjects (7.7%) in the once a month or less group, 5,322 subjects (5.9%) in the once a week group, 6,508 subjects (7.2%) in the 2 or 3 times a week group, 1,715 subjects (1.9%) in the almost every day group, and 16,400 subjects (18.1%) in the missing group. The percentage of subjects who reported the incidence of falls was 7.7% in the not participating group, 5.2% in the once a month or less group, 4.3% in the once a week group, 4.4% in the 2 or 3 times a week group, 3.4% in the almost every day group, and 8.1% in the missing group. The univariate model showed that, with the not participating subjects as a reference, the OR for subjects with once a month or less participation was significantly lower at 0.66 (95% CI 0.59–0.73) and was even lower for the subjects with once a week participation at 0.55 (95% CI 0.48–0.62); for subjects with 2 or 3 times a week participation, the OR was lower at 0.55 (95% CI 0.49–0.62), and for subjects with almost every day participation OR was at 0.43 (95% CI 0.33–0.56).

Table 3 shows the OR and 95% CI for the incidence of falls associated with the frequency of participation in a sport organization, in the adjusted models. After adjusting for base characteristics (model 1) and setting the not participating subjects as the reference, the OR for the subjects with once a week participation was significantly lower at 0.66 (95% CI 0.57–0.75) and was even lower for those with 2 or 3 times a week participation at 0.63 (95% CI 0.55–0.71), and the OR for subject with almost every day participation was at 0.49 (95% CI 0.38–0.64). Similar results were observed when we added physical traits, depression, and life habits to the covariates in model 1 (model 2, once a week: OR = 0.82, 95% CI 0.71–0.94, 2 or 3 times a week: OR = 0.81, 95% CI 0.71–0.92, and almost every day: OR = 0.66, 95% CI 0.51–0.87) and added environmental characteristics to the covariates in model 2 (model 3, once a week: OR = 0.82, 95% CI 0.72–0.95, 2 or 3 times a week: OR = 0.81, 95% CI 0.72–0.92, and almost every day: OR = 0.67, 95% CI 0.52–0.88). This indicates that those who participated in a sport organization at least once a week had approximately 20% lower incidence of falls than those who did not participate at all. Moreover, lower incidence of falls was associated with more frequent participation in a sport organization.

### 3.2. Fall-Related Factors and Fall Incidence

The univariate models showed that older age, low educational status, low annual equivalent income, having current medical history, low physical ability, depression, <30 min walking time, once a week or less frequency of outings, not having parks or foot paths suitable for exercise or walking, having locations difficult for walking close to home, and living in the local government were each associated with the incidence of falls (Table 1). In the fully adjusted model 3, older age, male gender, low educational status, having current medical history, low physical ability, depression, less than 30-minute walking time, having locations difficult for walking close to home, and living in the local government were each associated with the incidence of falls. However, no significant association was observed between the incidence of falls and annual equivalent income, frequency of outings, and availability of parks or foot paths suitable for exercise or walking after adding all covariates in the logistic regression model (Table 3).

## 4. Discussion

The main findings of this study were as follows: (1) even after controlling for 10 individual factors, including age, amount of physical activity, and depression, and 3 environmental factors such as habitable population density, the risk of falls was approximately ≥20% lower in people who participated in sport organizations once a week than people who did not participate in sport organizations; (2) lower incidence of falls was associated with more frequent participation in a sport organization; and (3) the influence of sex and socioeconomic factors on falls was found to be different from the results of the previous studies.

First, this study sought to verify whether participating in sport organizations was linked to incidence of falls even after statistically controlling for multiple individual and environmental factors. The results of models 1–3 showed that people who participated in sport organizations at least once a week were less likely to fall compared to people who did not. This is thought to be through direct effects such as improvement in strength and balance [19, 20] that are associated with lower risk of falls [15]. It has been reported that exercising at least once per week for at least 2 hours is necessary in fall-prevention programs that have been shown to be effective [16]. In other words, it is highly likely that people who fulfill these conditions by participating in sport organizations instead of specialized fall-prevention programs fall less frequently.

The result shows that lower incidence of falls was associated with more frequent participation in a sport organization. A person with a higher participation frequency would demonstrate greater physical activity and exercise at a higher intensity during a session. In addition, Sherrington et al. mentioned that increasing the total hours of the exercise program to over 50 hours would lower the incidence of falls in older people [17]. Therefore, even if the period of participation in a sports organization is shorter, some participants with more participation frequency would satisfy this condition or requirement. However, those who participate in an organization often participate for a long duration. For that reason, the participation could be over 50 hours in total even if the frequency is not necessarily once or more a week; however, from a long-term standpoint, the exercise requirement for prevention is considered as secured. Therefore, not only the frequency but also the duration of one session and the exercise intensity are important.

On the other hand, previous studies have reported that the injury by falls causes declined physical functioning [2, 4]. It is also possible that the results at this time indicate an apparent relationship of falls with increased physical activity and not with participation in a sports organization. Thus, we have conducted analyses with adjustment for walking in minutes/day and the frequency of outing as an index of physical activity in this study. The results showed that the OR of the frequency of participation in a sport organization increased significantly from 0.66 to 0.82 for those with once a week participation, from 0.63 to 0.81 for 2 or 3 times a week, and from 0.49 to 0.66 for those with almost every day participation. Street et al. reported that participating in sport organizations not only influences physiological health by increasing the amount of physical activity but also works via social networks and social support [21]. It is therefore also possible that participation in sports organizations indirectly protects health through social support and social networks. Further, a 4-year follow-up cohort study on 11,581 people by Kanamori et al. suggested an indirect effect whereby, among people with the same exercise frequency, those who did not participate in sport organizations had a 1.33-fold higher hazard ratio for being certified to require long-term care compared to those who did [24]. The above indicates that people who participate in sport organizations get more physical activity than people who do not, which leads to less likelihood of falling through direct effects such as increased strength and balance as well as indirect effects such as the intervention of social networks and social support.

Next, we will discuss the relationship between environmental factors and the results of this analysis. Previous studies have reported that participation rates in sport organizations are higher in urban areas [35] and that exercise frequency is greater among people who live near parks or other areas suitable for exercise [36]. However, it is possible that these relationships are only apparent due to confounding factors. Therefore, we controlled for habitable population density and variables regarding the neighborhood environment. The results showed that, in the built environment, a significant relationship did not exist with the presence of facilities such as parks and the OR for falls of people living in communities without slopes or steps was low at 0.74. Further, in the community characteristics observed through population density, the OR of falls was significantly higher in rural areas (1.43) than in urban areas. There was little change in the OR for participation in sport organizations even compared to models 2 and 3 including these environmental variables, which shows that the confounding effect of these environmental characteristics is small. The results suggest that increasing the number of participants in sport organizations could reduce falls even after considering for environmental and individual factors such as depression and motor ability. This supports the significance of further research into population strategies via community initiatives such as policies that promote participation in sport organizations (participatory organizations), which could serve as fall-prevention measures to complement or substitute for primary or secondary prevention. Tinetti et al. [37] conducted a large interventional study in 2 communities with around 100,000 people aged ≥70 years. In addition to individual interventions, they used soft methods such as placing posters on buses and other means of public transport, handing out pamphlets, making appeals through media such as radio and television, and holding seminars. Through this, they found that fall-related injuries and medical expenses declined in the intervention community compared to the control community. Further research on fall-prevention policies that employ population strategies to promote participation in sport organizations is needed.

Many previous studies [15, 27, 38] have examined factors related to falls, particularly a systematic review of 74 cohort studies by Deandrea et al. [9]. This study obtained similar results to past research regarding age, physical characteristics, and mental characteristics. However, we obtained different results regarding gender, annual equivalent income, and frequency of outings. One very likely reason for these differences was that we considered and controlled for variables simultaneously. For example, regarding the link between gender and the incidence of falls, Deandrea et al. reported that nearly all the studies they reviewed found being female is associated with an increased risk of falls [9], with the same being reported in other past studies [38]. However, while many of these studies had sample sizes in the thousands, none of the analyses were controlled for depression. This study found a significant relationship between gender and the incidence of falls both in the univariate analysis and in model 1, which controlled for age and gender. However, in model 2, which controlled for psychological factors including depression, being female was found to have a significantly lower OR with regard to the incidence of falls compared to being male. Women are at higher risk of depression than men [39–41], and depression has been shown to be a risk factor for falls [13, 14, 42]. In other words, controlling for depression, which was not done in previous studies, produces results indicating that women are less likely to fall than men.

A study from South Australia on the relationship of history of falls with socioeconomic factors reported that many people with at least a university degree had not experienced a fall in the past 1 year [43]. Moreover, Fabre et al. pointed to several pieces of evidence in reporting that many years of education and higher incomes were linked to a lower risk of falling [27]. Matsuda in an analysis of about 30,000 community-dwelling older people in Japan also reported higher ratios of people who had experienced a fall among subjects with low levels of education and income [29]. Examination of these studies, however, reveals that few simultaneously included both years of education and income in their analyses. The univariate analysis in this study, as well as the multivariate analyses in models 1 and 2 that simultaneously included years of education and annual equivalent income, showed significantly higher likelihood of falling in both people with few years of education and those with low annual equivalent incomes. People of low socioeconomic status become depressed more easily, get less physical activity [44, 45], and often exhibit shorter walking times [29]. Since people with depression and lower levels of physical activity are at risk of falling [13, 14, 42], it is possible that socioeconomic factors are linked to falls via differences in the incidence of depression and amount of physical activity. After controlling for depression, the amount of physical activity, and other individual characteristics in model 2, the OR of the socioeconomic factors declined significantly, from 1.86 to 1.52 for <6 years of education and from 1.29 to 1.08 for equivalent annual income <1.5 million yen. In other words, while factors such as depression and physical activity were found to participate in the relationship between socioeconomic factors and the incidence of falls in this study, the existence of other routes is suggested. In model 3, which controlled for environmental factors, only the annual equivalent income declined significantly, suggesting that years of education are more strongly linked to falls than to income.

While the advantages of this study are that it used a relatively large amount of data and controlled for a large number of variables, it has several limitations. First, since this was a cross-sectional analysis, we merely showed that a relationship exists between participation in sport organizations and the incidence of falls; we did not eliminate the effect of an opposite causal relationship, whereby falls prevent people from participating in sport organizations. Further, the influence of the subjects' objective motor functions or unknown confounding factors was not eliminated. In the future, we hope that a longitudinal research using 2 time points will be pursued to determine a causal relationship. Second, the environmental variables in this study were obtained from self-administered questionnaires. It is becoming increasingly possible to obtain objective information on slopes, differences in elevation, and other features from geographic information systems (GIS); it would be desirable to use such information in future investigations. Third, in this study, we did not investigate the duration of one session and intensity of the activity. Thus, if the promotion of participation in sport organizations is pursued as a population strategy to prevent falls, we also need to consider the influence of the duration and intensity of the activity, in addition to the frequency of participation in sports. Fourth, the results of population strategies must eventually be verified through community-intervention studies.

## 5. Conclusion

This study analyzed the data of 90,610 community-dwelling older people individuals. Even after adjusting for multiple factors it was found that the risk of falls was approximately ≥20% lower in people who participated in sport organizations at least once per week than those who did not participate at all. This suggests that primary prevention through population strategies such as policies that promote participation in sport organizations could be beneficial as measures for preventing falls in the community-dwelling older people. In the future, longitudinal analyses to elucidate causal relationships and community-intervention studies should be pursued.

## Figures and Tables

**Table 1:** Characteristics and univariate associations of falls with covariates.

	Total	Fallers		Crude OR (95% CI)
	*n*	*n*	(%)	
*N*	90,610	6,391		
Age (years)				
65–69	26,425	1,185	(4.5)	1.00
70–74	26,523	1,504	(5.7)	1.28 (1.18–1.38)**
75–79	20,176	1,615	(8.0)	1.85 (1.72–2.00)**
80–84	11,773	1,310	(11.1)	2.67 (2.46–2.89)**
≥85	5,713	777	(13.6)	3.35 (3.05–3.69)**
Sex				
Male	41,912	2,893	(6.9)	1.00
Female	48,698	3,498	(7.2)	1.04 (0.99–1.10)
Educational attainment (years)				
≥13	15,282	792	(5.2)	1.00
10–12	29,845	1,679	(5.6)	1.09 (1.00–1.19)
6–9	39,259	3,247	(8.3)	1.65 (1.52–1.79)**
<6	2,268	341	(15.0)	3.24 (2.83–3.71)**
Missing	3,956	332	(8.4)	1.68 (1.47–1.91)**
Equivalent income (yen)				
≥250	23,074	1,280	(5.5)	1.00
1,500,000–2,500,000	29,696	1,798	(6.1)	1.10 (1.02–1.18)*
<1,500,000	22,045	1,865	(8.5)	1.57 (1.46–1.69)**
Missing	15,795	1,448	(9.2)	1.72 (1.59–1.86)**
Present illness related to falls^§^				
No	40,739	2,265	(5.6)	1.00
Yes	28,244	3,193	(11.3)	2.17 (2.05–2.29)**
Missing	21,627	933	(4.3)	0.77 (0.71–0.83)**
Physical ability				
Go upstairs without holding rail or wall				
Yes	74,970	3,933	(5.2)	1.00
No	14,603	2,354	(16.1)	2.38 (2.26–2.51)**
Missing	1,037	104	(10.0)	2.23 (1.79–2.77)**
Stand up from the chair without any aids				
Yes	54,696	2,592	(4.7)	1.00
No	34,991	3,707	(10.6)	3.47 (3.29–3.67)**
Missing	923	92	(10.0)	2.01 (1.64–2.47)**
Depression				
No	53,912	2,516	(4.7)	1.00
Mild	15,509	1,624	(10.5)	2.39 (2.24–2.55)**
Moderate to severe	5,144	880	(17.1)	4.22 (3.88–4.58)**
Missing	16,045	1,371	(8.5)	1.91 (1.78–2.04)**
Walking in min/day				
≥30	58,993	3,481	(5.9)	1.00
<30	28,627	2,623	(9.2)	1.61 (1.53–1.70)**
Missing	2,990	287	(9.6)	1.69 (1.49–1.92)**
Frequency of outings				
Almost every day	46,063	2,546	(5.5)	1.00
2-3 times a week	25,421	1,845	(7.3)	1.34 (1.26–1.42)**
Once a week or less	15,181	1,633	(10.8)	2.06 (1.93–2.20)**
Missing	3,945	367	(9.3)	1.75 (1.56–1.97)**
Neighborhood built environment				
Parks and walkways for exercise				
Yes	61,667	3,937	(6.4)	1.00
No	24,071	1,908	(7.9)	1.26 (1.19–1.34)**
Missing	4,872	546	(11.2)	1.85 (1.68–2.03)**
Difficult to walk on a slope or steps				
Yes	35,787	3,106	(8.7)	1.00
No	50,321	2,814	(5.6)	0.62 (0.59–0.66)**
Missing	4,502	471	(10.5)	1.23 (1.11–1.36)**
Population density (person per square kilometer)				
Urban	36,878	2,025	(5.5)	1.00
Semiurban	20,327	1,429	(7.0)	1.30 (1.21–1.40)**
Rural	33,405	2,937	(8.8)	1.66 (1.56–1.76)**

OR: odds ratio; CI: confidence interval.

^§^Stroke, osteoporosis, joint disease/neuralgia, injury/fracture, mental illness, impaired vision, and impaired hearing.

**P < 0.01, *P < 0.05.

**Table 2:** Univariate associations of falls with participation in sport organizations.

	Total	Fallers		Crude OR (95% CI)
	*n*	*n*	(%)	
*N*	90,610	6,391		
Frequency of participation in sport organizations				
Not participating	53,645	4,121	(7.7)	1.00
Once a month or less	7,020	363	(5.2)	0.66 (0.59–0.73)**
Once a week	5,322	231	(4.3)	0.55 (0.48–0.62)**
2 or 3 times a week	6,508	286	(4.4)	0.55 (0.49–0.62)**
Almost every day	1,715	59	(3.4)	0.43 (0.33–0.56)**
Missing	16,400	1,331	(8.1)	1.06 (1.00–1.13)

OR: odds ratio; CI: confidence interval.

**P < 0.01.

**Table 3:** Multivariate adjusted OR and 95% CI for the associations of falls.

	Model 1	Model 2	Model 3
	OR (95% CI)	OR (95% CI)	OR (95% CI)
Frequency of participation in sport organizations			
Not participating	1.00	1.00	1.00
Once a month or less	0.77 (0.69–0.87)**	0.94 (0.84–1.05)	0.91 (0.81–1.02)
Once a week	0.66 (0.57–0.75)**	0.82 (0.71–0.94)**	0.82 (0.72–0.95)**
2 or 3 times a week	0.63 (0.56–0.71)**	0.81 (0.71–0.92)**	0.81 (0.72–0.92)**
Almost every day	0.49 (0.38–0.64)**	0.66 (0.51–0.87)**	0.67 (0.52–0.88)**
Age (years)			
65–69	1.00	1.00	1.00
70–74	1.23 (1.13–1.33)**	1.12 (1.03–1.21)*	1.10 (1.01–1.19)*
75–79	1.68 (1.55–1.82)**	1.29 (1.19–1.40)**	1.25 (1.16–1.36)**
80–84	2.32 (2.13–2.52)**	1.57 (1.43–1.71)**	1.50 (1.38–1.64)**
≥85	2.74 (2.48–3.02)**	1.65 (1.49–1.83)**	1.57 (1.42–1.75)**
Sex			
Male	1.00	1.00	1.00
Female	0.95 (0.91–1.01)	0.79 (0.75–0.84)**	0.79 (0.75–0.84)**
Educational attainment (years)			
≥13	1.00	1.00	1.00
10–12	1.04 (0.95–1.13)	0.99 (0.91–1.08)	0.98 (0.90–1.07)
6–9	1.37 (1.26–1.49)**	1.24 (1.14–1.35)**	1.22 (1.12–1.33)**
<6	1.86 (1.61–2.14)**	1.52 (1.31–1.75)**	1.47 (1.27–1.71)**
Equivalent income (yen)			
≥250	1.00	1.00	1.00
1,500,000–2,500,000	1.07 (0.99–1.15)	1.01 (0.94–1.09)	1.00 (0.92–1.08)
<1,500,000	1.29 (1.20–1.40)**	1.08 (1.00–1.17)*	1.02 (0.94–1.10)
Present illness related to falls^§^			
No		1.00	1.00
Yes		1.59 (1.50–1.69)**	1.57 (1.48–1.67)**
Physical ability			
Go upstairs without holding rail or wall			
Yes		1.00	1.00
No		1.45 (1.36–1.53)**	1.44 (1.36–1.53)**
Stand up from the chair without any aids			
Yes		1.00	1.00
No		2.02 (1.89–2.14)**	1.98 (1.86–2.11)**
Depression			
No		1.00	1.00
Mild		1.79 (1.68–1.92)**	1.78 (1.66–1.90)**
Moderate to severe		2.72 (2.49–2.97)**	2.68 (2.45–2.93)**
Walking in min/day			
≥30		1.00	1.00
<30		1.09 (1.03–1.15)**	1.09 (1.03–1.16)**
Frequency of outings			
Almost every day		1.00	1.00
2-3 times a week		0.99 (0.93–1.06)	0.96 (0.90–1.03)
Once a week or less		1.11 (1.04–1.20)**	1.03 (0.96–1.11)
Neighborhood built environment			
Parks and walkways for exercise			
Yes			1.00
No			1.03 (0.97–1.09)
Difficult to walk on a slope or steps			
Yes			1.00
No			0.74 (0.70–0.79)**
Population density (people per square kilometer)			
Urban			1.00
Semiurban			1.23 (1.14–1.32)**
Rural			1.43 (1.35–1.53)**

OR: odds ratio; CI: confidence interval; ^§^stroke, osteoporosis, joint disease/neuralgia, injury/fracture, mental illness, impaired vision, and impaired hearing.

Model 1 is adjusted for age, sex, educational attainment, and equivalent income.

Model 2 is adjusted for the covariates in Model 1 plus physical ability, depression, walking in minute/day, and frequency of outings.

Model 3 is adjusted for the covariates in Model 2 plus neighborhood built environment and population density.

**P < 0.01, *P < 0.05.
